# Application of thermosonication for guava juice processing: Impacts on bioactive, microbial, enzymatic and quality attributes

**DOI:** 10.1016/j.ultsonch.2023.106595

**Published:** 2023-09-07

**Authors:** Baldev Singh Kalsi, Sandhya Singh, Mohammed Shafiq Alam, Surekha Bhatia

**Affiliations:** Department of Processing & Food Engineering, Punjab Agricultural University, Ludhiana, Punjab, India

**Keywords:** Guava juice, Thermosonication, Pasteurization, Microbial inactivation, Enzymes, Bioactive compounds

## Abstract

The present work explores different conditions of thermosonication (TS) processing that would ensure microbiological and enzymatic safety for guava juice while simultaneously maximizing the preservation of its quality attributes. The guava juice was subjected to TS treatment (frequency: 40 kHz; power: 200 W; Temperature: 40, 60, and 80 °C; Time: 2, 6 and 10 min) and was compared with fresh and pasteurized (90 °C/60 s) juice samples. The objectives of the research work were to determine the effect of thermosonication on the quality attributes such as total soluble solids (TSS), pH, titratable acidity, cloud value, color attributes, total phenolic contents, total flavonoid contents, antioxidant activity, ascorbic acid levels, enzymatic, microbiological, and sensory properties. The thermosonicated and pasteurized samples showed no significant (p > 0.05) changes in pH, total soluble solids, and titratable acidity. TS improved the cloud value and color attributes. Furthermore, TS enhanced total phenols (10 to17%), flavonoids (5 to 25%), antioxidant activity (10.45% to 14.55%) and retention of ascorbic acid (61.98–83.32%) relative to control. Thermosonicated sample at 80 °C/10 min gives the maximum inactivation of Pectin methyl esterase (PME), Peroxidase (POD) and Polyphenol oxidase (PPO) enzymes. While both thermosonication and pasteurization drastically decreased the microbial count to undetectable levels, only TS exhibited modest improvement in sensory qualities. The results demonstrated that TS can enhance the overall safety, quality, and commercial viability of guava juice as a practical substitute to pasteurization.

## Highlights

• Thermosonication had non-significant effect on the physicochemical properties of juice.
• Thermosonication promoted higher retention of ascorbic acid in comparison to thermal treatment.
• Thermosonication enhanced the bioactive compounds.
• Thermosonication significantly inactivated the microbial and enzymatic load.
• The findings showed that thermosonication has the potential for industrial applications.

## 1 Introduction

Guava (*Psidium guajava* L.), a tropical fruit belongs to the family of Myrtaceae. It is often regarded as a superfruit due to its high content of vitamin A and C, polysaturated fatty acids and dietry fibres [21]. The content of vitamin C in guava fruit is about four times more than that of an orange fruit. Moreover, guava fruit provides an appropriate amount of dietary minerals, potassium, magnesium, and a comprehensive, low-calorie profile of important nutrients [24]. Guava has been proven to have a variety of biological and pharmacological qualities, including antioxidant, anti-inflammatory, immunomodulatory, antibacterial, diabetic, and anticancer effects [15]. Guava marketing and storage are tough because of its short shelf life [6]. As a result, processing of guava fruit into other sort of goods such as juices, puree, pulp and jam that can increase shelf life and quality is necessary. Fruit juices have lately been popular in the market as people choose natural juices over beverages that have caffeine for example tea, coffee, and carbonated drinks [18]. Fruit juices serves as an effective processing method with the advantage of delivering highly concentrated nutrients [35]. Thermal pasteurization or sterilization has been widely employed for extending shelf life of juices by inactivating the loads of microbes and enzymes. However, these traditional preservation techniques have shown negative impacts on nutritive and sensorial values of the juices [16]. As a result, an alternative to traditional techniques has been investigated in order to meet present demand while also enhancing quality and safety for consumers.

Ultrasound processing/ sonication have been identified as a new non-thermal way of increasing juice quality. The high power ultrasound at low frequencies varying from 20 to 100 kHz, when propagates in liquid promotes the generation, expansion and explosion of bubbles. The explosion of bubbles generates a local spot of high temperature and pressure and this whole phenomenon is known as cavitation [43]. These elements have a role in mediating numerous kinds of chemical processes and cell disturbances leading to microbial cell death [29]. Considering the industrial aspect, cellular alteration at microscopic level caused by ultrasound can lessen the requirement for the addition of stabilizers to the juice [38]. These changes improve the juices' sensory quality by enhancing the stability, improving the color, increasing turbidity and apparent viscosity, and delaying pulp sedimentation [22]. Furthermore, sonication has proven to be a beneficial alternative to thermal treatment in terms of low energy input, minimum processing, and shorter processing times [1]. Ultrasound may be used in conjunction with other techniques to improve microbial inactivation efficiency, and many researches have been conducted combining sonication with either temperature or pressure and temperature. Recently, it was proven that combining ultrasound and thermal processing techniques helped increasing juice safety [19].

Thermosonication (TS), known by another name as ultrasonic-assisted heat treatment, has gained increasing attention and application in the processing of liquid products like juices, milk, beverages and others, due to its capability to inactivate the enzymes and microorganisms with the least possible loss of quality in comparison to traditional thermal treatments [9], [26], [45], [53]. TS of tangor juice has been found to be significantly more effective in preserving/enhancing the levels of bioactive compounds compared to traditional pasteurization [7]. TS has shown substantial enzyme inactivation (lipoxygenase (LOX) and peroxidase (POD) for almond (Prunus dulcis) milk while enhancing the bioavailability of total phenolic, flavonols, flavonoids, condensed tannin contents, and antioxidant activity when compared to pasteurization [25]. TS not only better preserves sensory and physicochemical characteristics than traditional pasteurization but has extended the shelf life of pulque (a non-dairy, fermented, and alcoholic beverage) by up to 24 days [4]. Soft cheese prepared with thermosonicated mik has shown enhanced color parameters (higher lightness (L*), and whiteness index (WI) milk when compared to conventional pastrurized milk [47]. TS of orange juice whey drinks showed better functional properties (anti-hypertensive and anti-diabetic activities) and rheological paramters (consistency and higher homogeneity) in comparison to short-time heat-treatment (90 °C/20 s) [32]. Thus, thermosonication is regarded as an excellent substitute for traditional thermal procedures (pasteurization) for inactivating enzymes and bacteria while preserving the essential quality characteristics of liquid products.

Guava juice is well-liked by customers due to its delicious flavour and full nutritional profile [41], [44]. Thus, ensuring the preservation of its sensory characteristics, microbial safety, and nutritional properties is of paramount importance in its processing. While various methods have been employed to process guava juice [10], [21], there is a notable gap in research regarding the application of thermosonication. Specifically, limited information is available regarding the effects of thermosonication on microbial reduction (including Total Plate Count and Yeast and Mold Count), pectin methylesterase (PME) inactivation, cloudiness behavior, and sensory properties of freshly prepared guava juice. Furthermore, prior researches have demonstrated the use of ultrasound at a frequency of 20 kHz during thermosonication [13], [39]. However, using a higher frequency of 40 kHz with varied combinations of exposure temperature and time is anticipated to result in localized high pressure and energy and that would have resulted in effective sterilization at local zones. So the current study was planned with the objective of tracking the influence of thermosonication at a frequency of 40 kHz on microbial and enzyme inactivation of fresh guava juice in addition to evaluating the impact of thermosonication on additional critical quality characteristics such as physicochemical, bioactive, color, and sensory properties. Further, a conventional thermal treatment was given to guava juice for comparison purposes. This study is expected to raise awareness about the potential benefits of thermosonication to preserve the overall quality attributes of guava juice.

## 2 Materials and methods

### 2.1 Guava juice preparation

The harvesting of fully matured guava fruits (*Psidium guajava*) was done at the orchards of Punjab Agricultural University, India and fruits were brought to Food Engineering lab. Each guava was washed, drained, peeled, and cut lengthwise into pieces using a sterilised stainless steel knife after the defective fruit was screened. The juice was extracted from the guava fruit slices using a mechanised juice extractor (Inalsa, India). To ensure a uniform consistency, the juice was filtered through a sterile cotton cloth.

### 2.2 Thermosonication and pasteurization treatment

Thermosonication was done using an ultrasound bath (Helix Bioscience Ultrasonic, India) at a constant frequency of 40 kHz and full power of 200 W. About 50 ml of guava juice samples were thermosonicated in bath type sonicator for three distinct temperatures: 40, 60 and 80 °C and at each temperature, samples were held for three different times: 2, 6 and 10 min. Trials were conducted in an area that was dark to eliminate interference from light. Samples of guava fruit juices were pasteurized in lab scale pasteurizer at 90 °C for 60 s [29]. After treatment, juice samples were filled in sterilized containers and stored at 4 °C until further analysis. The scheme of different treatments was as UGJ: Untreated guava juice, PGJ: Pasteurized guava juice, TS40-2, TS40-6, TS40-10: Thermosonicaton of guava juice at 40 °C for 2 min, 6 min and 10 min; TS60-2, TS60-6, TS60-10: Thermosonicaton of guava juice at 60 °C for 2 min, 6 min and 10 min; TS80-2, TS80-6, TS80-10: Thermosonicaton of guava juice at 80 °C for 2 min, 6 min and 10 min. Every treatment was performed in triplicate.

### 2.3 Total soluble solids (TSS), pH, and titratable acidity (TA)

A hand refractometer (Erma, Japan) was used to estimate TSS at 25 ± 1 °C and was represented in °Brix units. The measurement of the pH value of guava juice was measured using a digital pH metre (Elico, India) at 25 ± 1 °C. For evaluating the titratable acidity (TA), the standard visual titration method was used [36]. The guava juice samples (10 ml) were diluted with 90 ml distilled water and an aliquot of 10 ml was titrated with standardised 0.1 N sodium hydroxide to a brightly pink colored tone (color holding ought to last longer than fifteen seconds) in the presence of a indicator (phenolphthalein). To measure the TA (%) of juice, the sodium hydroxide used in the titration procedure was transformed to grams of citric acid per 100 ml of juice using equation (1).

(1) $$TA\left(\%\right)=\frac{T\times N\times V_{1}\times E}{V\times V_{2}\times 1000}\times 100$$

where T is the titre volume (ml), N is the normality of standard NaOH, V_1_ is the volume made up (ml), E is the equivalent weight of acid, V_2_ is the aliquot volume (ml), and V is the volume of sample taken (ml).

### 2.4 Cloud value

For estimating the cloud value of guava juice, supernatant was obtained after centrifuging (3000 rpm for 10 min) the juice samples of 5 ml. Finally, the absorbance of this supernatant was measure using a spectrophotometer (Model:UV-2601 UV/VIS Double Beam, Rayleigh, China) at a wavelength of 660 nm with distilled water serving as a blank [5].

### 2.5 Color analysis

In guava juice, the color values were evaluated by means of a colorimeter (Konica Minolta CR-10 color reader, Japan) using the method of [17]. The color values were expressed in form of L*, a* and b* where value of L* is measure of lightness and vary from 0 (black) to 100 (perfect white)*,* value of a* is either negative (greenness) or positive (redness) and value of b* are also either positive (yellowness) or negative (blueness). These were changed into Chroma and hue angle using following equations 2 and 3.

Chroma (C*) =${\left(a^{{*}^{2}}+b^{{*}^{2}}\right)}^{1/2}$ (2)

Hue angle (h*) = $\tan^{-1}\left(\frac{b^{*}}{a^{*}}\right)$when a* >0 and b* >0 (3)

(h*) = ${180^{{}^{\circ}}\;+\tan}^{-1}\left(\frac{b^{*}}{a^{*}}\right)$when a* <0 and b* >0 or b* <0

(h*) = ${360^{{}^{\circ}}+\tan}^{-1}\left(\frac{b^{*}}{a^{*}}\right)$when a* >0 and b* <0

### 2.6 Ascorbic acid content

The 2,6-Dichlorophenol-Indophenol dye (DID) visual titration method was used to determine the ascorbic acid level of guava juice [16]. A known quantity of juice (10 ml) is put into a 100 ml volumetric flask and volume make up was done using 0.4% oxalic acid solution. After filtration through a whatsman No. 4 filter paper, the titration of an aliquot of 10 ml was done against the standardized dye solution of DID to an end-point of faint pink color persisting for at least 15 s. Results were calculation using equation (5) and expressed in form of mg/100 ml of each guava juice sample. The following formula is used to compute the dye factor in mg of ascorbic acid per mL:

(4) $$\mathrm{Dye}\;factor=\frac{0.5}{\mathrm{titre}}$$

(5) $$\mathrm{Ascorbic}\;acid\left(mg/100ml\right)=\frac{\mathrm{Value}\;of\;titre\;in\;ml\times Dye\;factor\times Volume\;made\;up}{\mathrm{Aliquot}\;taken\times Volume\;of\;sample\;taken}\times 100$$

### 2.7 Total flavonoids content

The spectrophotometric approach was used to determine the total flavonoids content of guava juice as described by Nayak et al. [30]. A known amount of extracted juice sample (50 µl) was mixed in 30 µl solution of sodium nitrite (5%) and 60 µl of AlCl_3_ (10%) followed by addition of 200 µl of 1.0 M Sodium hydroxide solution. Absorbance was measured by spectrophotometer at 510 nm and catechin was taken as standard. The results were presented as mg of catechin equivalents per 100 ml of the sample.

### 2.8 Total phenolic content (TPC)

TPC of guava juice was measured by using the Folin–Ciocalteu method described by Kalsi et al. [16]. Briefly, a 1 ml portion of properly diluted 80% methanol extracts was introduced into a 25 ml volumetric flask containing 9 ml of distilled water. Following this, 0.5 ml of the Folin–Ciocalteu phenol reagent was introduced to the mixture and vigorously agitated. After a 5-minute interval, 5 ml of a 5% Na_2_CO_3_ solution was incorporated while stirring. The solution was promptly diluted to a final volume of 25 ml using distilled water and thoroughly mixed. It was then allowed to sit for 60 min before the absorbance was measured at 750 nm in comparison to the control sample prepared using distilled water. The total phenolics of juice specimens were calculated using a linear equation that was developed from the calibration curve drawn employing gallic acid as the standard of reference and the results were shown as mg of Gallic acid equivalent (GAE)/100 ml of guava juice.

### 2.9 Antioxidant capacity

For estimating antioxidant activity of guava juice, 2, 2-diphenyl-1pikryl-hydrazyl (DPPH) radical method [30] was used. The method involved mixing of a known amount of juice aliquot (2 ml) with 2 ml of DPPH solution (0.2 nM) followed by incubating the mixture in a dark place for half an hour. The proton donating activity decreased the absorbance which was recorded at a wavelength of 517 nm and DPPH inhibition % was calculated using following formula:

Antioxidant activity % $=\frac{A_{0}-A_{s}}{A_{0}}\times 100$

where, A_o_ and A_s_ stands for the absorbance of control and sample, respectively.

### 2.10 PME, POD, and PPO residual activities

Pectin methyl esterase (PME), Peroxidase (POD) and Polyphenol oxidase (PPO) residual activity were determined using method of Dars et al. [11]. The samples of gauva juice were centrifuged at 8000*g* for 15 min at 4 °C for the enzyme activity assay. For determining PME, supernatant (10 ml) was added to 40 ml solution of pectin (1 g/100 ml) which conatins 0.15 mol/L NaCl. The solution's pH was changed to 7.7 by incorporating 0.05 mol/L NaOH, and the amount of time involved was noted. One unit of PME is defined as the amount of enzyme that released 1 μmol of carboxyl groups in 1 min. For evaluating POD, mixing of 0.32 ml of 0.2 mol/L potassium phosphate buffer (pH 6.8), 0.32 ml of pyrogallol (5%) and 0.6 ml of H_2_O_2_ (0.147 mol/L) and recording the increase in absorbance at 420 nm within 3 min was done. With repect to PPO, 1.5 ml supernatant was mixed with 0.5 ml catechol (0.5 mol/L) and 3.0 ml potassium phosphate buffer (0.2 mol/L, pH 6.8). The absorbance was recorded at 410 nm within 3 min.The percent residual activity of PME, POD and PPO was calculated using following equation.

Residual activity (%)$=\frac{A_{t}}{A_{o}}\times 100$

Where: A_t_ and A_0_ represent the enzyme activity of treated and untreated samples, respectively.

### 2.11 Microbiological analysis

Total plate counts, as well as yeast and mold counts, were calculated in order to determine the microbiological safety of guava juice. Total bacteria were counted using the spreading plate methodology with plate count agar followed by incubation for 48 h (37 ± 1 °C). Similarly, yeast and mould were counted using potato dextrose agar and incubated for 120 h (25 ± 1 °C). Following incubation, the plates for growth were examined and number of colonies were counted and expressed as log (colony forming units/ml) of guava juice samples [16].

The microorganisms were counted in the untreated sample (control) and treated samples. Then the number of log reductions (*γ*) for microbial inactivation was calculated based on the untreated sample (N0) using following equation [32]

$$\gamma =\log_{10}\left(N0\right)-\;\log_{10}\left(Nf\right)$$

where *N0* is the untreated sample's viable microbe count and *Nf* is the treat sample's viable microbe count.

### 2.12 Sensory analysis

The examination of organoleptic qualities is critical for determining the acceptance of treated samples by consumers. A group of 20 (semi-trained) participants who were familiar with the fruit was created for the evaluation. Between sample assessments, panellists were given instructions to rinse their mouths with water. The whole assessment was done to compare the sensory qualities including color, taste, mouthfeel, and overall acceptability of guava juice samples that were treated to control one. The outcomes were documented using a nine-point hedonic scale (1 = very disliked and 9 = strongly liked) [16].

### 2.13 Statistical evaluation

The collected findings were evaluated with Minitab 19® software (Minitab Inc., State College, PA, USA) employing ANOVA at a 95% confidence level (p-value ≤ 0.05), and graphs were generated with Microsoft Word Excel 2007. The results were presented as mean ± SD.

## 3 Results and discussion

### 3.1 pH, total soluble solids (TSS) and titratable acidity

**Fig. 1:**
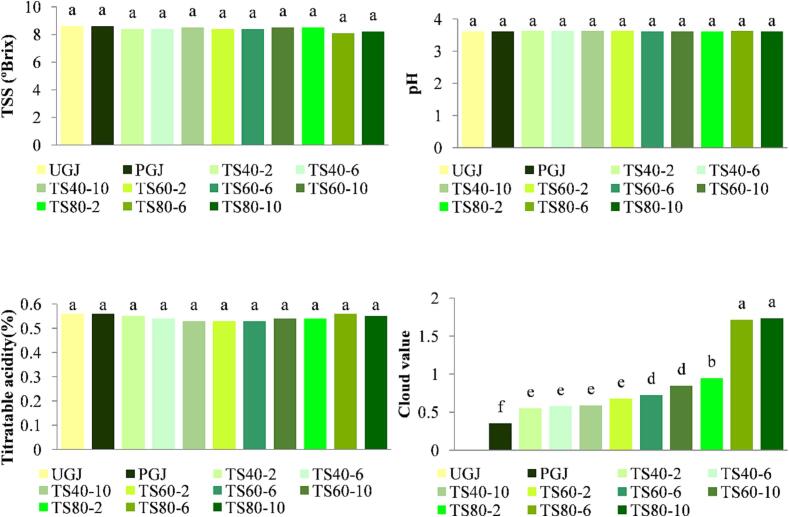
pH, total soluble solid, titratable acidity and cloud value of guava juice treated by different conditions of thermosonication. *Note*: Means ± SD, Bars with same superscript letters are non-significant at p < 0.05.

The effect of various processing treatments on the pH, total soluble solids of the guava juice are depicted in Fig. 1. The pH, total soluble solids, and titratable acidity of the untreated gauva juice (UGJ) was observed 3.61 ± 0.06, 8.6 ± 0.05 °Brix, 0.56 ± 0.02 %, respectively. Even following heat and thermosonication treatments, the pH level of guava juice did not alter substantially (p > 0.05). In a similar way, thermosonication and thermal treatment had no significant (p > 0.05) effect on total soluble solids or titratable acidity (p > 0.05). These findings might be explained by the fact that the energy level of ultrasound could not modify the structure associated with the aforementioned characteristics at the microscopic level [16]. The results of this study are in line with the non-significant effect of thermal treatment and thermosonication on the juices of blood fruit (*Haematocarpus validus*) and hog plum (*Spondias mombin* L.) [31], [35]. This indicates that thermosonication is a secured and potential tool as it didn’t cause any substantial changes in fundamental physicochemical parameters.

### 3.2 Cloud value

The combination of several elements such as pectin, lipids, cellulose, hemicellulose, and protein, as well as other particles, contributes to the value of cloud in apple juice. [1]. The cloud value ranged from 0.324 ± 0.15 to 1.734 ± 0.02 and temperature and processing time of thermosonication showed a significant effect (p < 0.05) on cloud value in comparison to cloud values of both fresh and pasteurized juice samples which is shown in Fig. 1. The sample thermosonicated at 80 °C for 10 min showed the highest cloud value with a value of 1.734. The results from this study are consistent with the strong influence of thermosonication on the cloud value of pomelo juice [8]. The rise in cloud value after thermosonication is possibly related to cavitation, which exerts a gradient of high-pressure and causes the surface area to increase, leading to the breakdown of big molecules, principally pectin together with other polysaccharides (cellulose hemicellulose), to smaller ones [16]. Moreover, the action of ultrasound is increased by the temperature because more transitory bubbles are burst at high temperatures and bigger molecules are broken [12].

### 3.3 Color analysis

The variation in the color of the guava juice treated by pasteurization and thermosonication is shown in Table 1. After thermosonication, value of lightness (l*), and yellowness (+b*) increased, while value of greenness (a*) decreased in contrast to untreated guava juice (UGJ). The results reported here are consistent with previous study on thermosonicated spinach juice by Manzoor et al. [26] which showed that this type of color variation was mostly connected to elevated processing temperatures, which resulted in lesser saturation and denser juice color. This increment can also be linked to cavitation which partially precipitated the unstable and suspended particles to form additional colored substance [31]. At higher temperature the value of a* was increased and value of b* was highly increased which might be linked to the degradation of chlorophylls during heat treatment [34]. Moreover, cavitation during thermosonication alters the color of juices because of enhanced chemical reactions, faster rate of diffusion and formation of polymers or decomposition of pigments [56]. Hue angle is a qualitative feature of color, with angles of 0, 90, 180, and 270 representing red, yellow-green, and blue colors, respectively [16]. In this study the hue angle of thermosonicated samples varied from 134.57° ±1.16 to 163.19° ±0.74. The thermosonication at 40 and 60 °C regardless of processing time enables hues of samples to steadily alter to the greener axis, while at high temperature of 80 °C, the hue of samples shifted to yellowish side with increasing temperature. Chroma is seen as a quantitative feature of color [16]. The thermosonicated sample TS40-10 showed the best chroma value (5.91). Similar results have been seen in thermosonicated spinach juice [26].

**Table 1:** Color attributes of guava juice treated by different conditions of thermosonication.

Treatment	L*	a*	b*	Hue angle	Chroma
UGJ	51.70 ± 1.52^c^	−3.81 ± 0.16^a^	1.61 ± 0.21^d^	157.06 ± 2.20^abc^	4.14 ± 0.21^f^
PGJ	59.45 ± 0.91^a^	−4.11 ± 0.05^b^	4.07 ± 0.87^a^	135.75 ± 1.92^f^	5.81 ± 0.54^abc^
TS40-2	51.01 ± 1.93^c^	−4.87 ± 0.04^cd^	1.61 ± 0.03^d^	161.70 ± 0.55^ab^	5.13 ± 0.03^de^
TS40-6	55.61 ± 1.60^abc^	−5.07 ± 0.17^d^	1.53 ± 0.02^d^	163.19 ± 0.74^a^	5.30 ± 0.16^bcde^
TS40-10	54.86 ± 1.08^abc^	−5.53 ± 0.19^b^	2.08 ± 0.06^cd^	159.37 ± 1.22^ab^	5.91 ± 0.16^a^
TS60-2	53.94 ± 0.29^bc^	−4.76 ± 0.13^cd^	1.63 ± 0.03^d^	161.09 ± 0.46^ab^	5.03 ± 0.13^e^
TS60-6	56.88 ± 0.10^ab^	−4.61 ± 0.10^bc^	2.54 ± 0.01^bc^	151.14 ± 0.56^cde^	5.26 ± 0.09^bcde^
TS60-10	59.63 ± 0.69^a^	−4.58 ± 0.15^bc^	2.68 ± 0.07^bc^	149.65 ± 1.51^df^	5.31 ± 0.10^abcde^
TS80-2	58.85 ± 2.39^ab^	−4.77 ± 0.09^cd^	2.11 ± 0.04^cd^	156.13 ± 0.58^bcd^	5.22 ± 0.08^cde^
TS80-6	59.60 ± 1.34^a^	−4.98 ± 0.20^cd^	3.11 ± 0.07^b^	148.00 ± 1.21^f^	5.87 ± 0.17^ab^
TS80-10	59.33 ± 2.25^a^	−3.98 ± 0.08^e^	4.04 ± 0.0.07^a^	134.57 ± 1.16^f^	5.67 ± 0.01^abcd^

*Note*: Means ± SD, Values with same superscript letters in the same column are non-significant at p < 0.05.

### 3.4 Ascorbic acid

Ascorbic acid is reported to be present in high concentrations in guava fruit and can be used to prevent cancers of the oesophagus, larynx, pancreas, and oral cavity [16]. Table 2 demonstrates the influence of thermosonication and thermal treatment on the ascorbic acid content of guava juice. 177.65 ± 0.80 and 70.80 mg/100 ml of ascorbic acid was reported in untreated fresh juice and pasteurized juice, respectively.

**Table 2:** Biochemical properties of guava juice treated by different conditions of thermosonication.

Treatment	Ascorbic acid (mg/100 ml)	Total phenol content (mg GAE/100 ml)	Flavonoids (mg CE/ 100 ml)	Antioxidant activity (% inhibition)
UGJ	177.65 ± 0.80^a^	230.71 ± 0.12^d^	49.71 ± 0.82^e^	80.93 ± 0.03^b^
PGJ	70.80 ± 0.85^g^	164.11 ± 0.85^f^	31.23 ± 0.33^i^	81.40 ± 0.88^b^
TS40-2	148.01 ± 0.14^b^	270.02 ± 0.43^a^	62.29 ± 0.56^a^	92.71 ± 0.68^a^
TS40-6	139.05 ± 0.77^bc^	252.04 ± 0.22^c^	59.60 ± 0.31^b^	91.59 ± 0.80^a^
TS40-10	135.21 ± 0.44^c^	249.10 ± 0.67^c^	58.48 ± 0.45^b^	90.52 ± 0.26^a^
TS60-2	132.07 ± 0.98^cd^	251.25 ± 0.25^c^	55.49 ± 1.28^c^	90.40 ± 0.30^a^
TS60-6	129.04 ± 0.01^cd^	264.25 ± 0.95^ab^	54.88 ± 0.31^c^	89.81 ± 0.67^a^
TS60-10	122.02 ± 0.41^de^	254.45 ± 0.06^bc^	52.27 ± 0.43^d^	89.39 ± 0.66^a^
TS80-2	118.03 ± 0.98^ef^	202.56 ± 0.21^e^	48.89 ± 1.36^f^	90.38 ± 0.96^a^
TS80-6	115.08 ± 0.14^ef^	193.85 ± 0.45^e^	42.34 ± 1.65^g^	90.31 ± 0.77^a^
TS80-10	110.10 ± 0.69^f^	174.55 ± 0.25^f^	38.26 ± 0.51^h^	89.50 ± 0.48^a^

*Note*: Means ± SD, Values with same superscript letters in the same column are non-significant at p < 0.05. GAE = Gallic acid equivalent.

Based on the values of the fresh guava juice, there was a more pronounced deterioration produced by the pasteurization treatment (60.14%) than by all TS treatments (in range of 16.68–38.02%). Among TS treated samples, the highest retention (83.32%) of ascorbic acid was observed in sample TS40-2 with a value of 148.01 ± 0.14 mg/100 ml, while the lowest retention (61.98 %) was observed in sample TS80-10 with a value of 110.10 ± 0.69. mg/ml. The more gentle the treatment, the better the vitamin C retention in juices since ascorbic acid is an unstable substance that decomposes easily in unfavorable circumstances [33]. With increasing temperatures and holding times during the TS treatments, more noticeable degradation of ascorbic acid was seen in present research of guava juice. Ascorbic acid degradation with increasing treatment temperatures may be related to extreme physical circumstances that resulted from the cavitational collapse of bubbles [50], [51], while the degradation with increasing holding time may be linked to oxidation reactions that are aided by the interaction of free radicals generated during sonication [37]. Overall, compared to traditional thermal treatment, the TS treatment offers more good ascorbic acid preservation in guava juice samples which are on par with the findings observed for the thermosonicated samples of orange juice whey drink [32].

### 3.5 Total phenolic content

The compounds known as phenols have a crucial function in human health by reducing the threat of various illnesses. In this investigation, total phenolic content in fresh and pasteurized juice was 230.71 ± 0.12 and 164.11 ± 0.85 mg GAE/100 ml, respectively as shown in Table 2. It was observed that phenolic content of the fresh juice was significantly (p < 0.05) higher than the pasteurized guava juice. Thermosonication at temperatures of 40 and 60 °C and holding time of 2 to 10 min significantly (*p* < 0.05) enhanced the level of total phenols by about 10 to17% when compared to fresh juice samples. The maximum value of total phenols (270.02 ± 0.43 mg GAE/100 ml) of guava juice was recorded for the thermosonicated sample TS40-2. This enhancement might be due to the reason that combination of temperature with cavitation effect of ultrasound breaked the macromolecules and released the bound form of the phenolic compounds [26]. Moreover, sonication generates hydroxyl radicals, which add to the aromatic ring of phenolic compounds and results in a rise of total phenolic level [16]. This rise corresponded to the findings of a study on the thermosonication of pomelo juice [8]. However, the thermosonication at 80 °C reduced the phenolic content by 12.20 to 24.30% which might be related to the depletion of phenolic content resulting from extreme temperatures [11]. The results reported here are consistent with those obtained in thermosonicated sugarcane juice at 80 °C [2]. Furthermore, as holding time increased, total phenolic content was slightly decreased at a certain temperature. This minor reduction in total phenolics might be attributed to free compounds being degenerated and the release of a quantity bound to the cell wall [48].

### 3.6 Total flavonoids content (TFC)

The effect of different processing treatments on the flavonids of guava juice has been shown in Table 2. It is evident that the total flavonoid content of pasteurized juice (31.23 ± 0.82 mg CE/100 ml) was significantly lower in contrast to that of raw juice (49.71 ± 0.82 mg CE/100 ml) and all thermosonicated samples (62.29 ± 0.56–38.26 ± 0.51 mg CE/100 ml). Thermosonication at temperatures of 40 and 60 °C and holding time of 2 to 10 min significantly (*p* < 0.05) enhanced the level of flavonoids by about 5 to 25% when compared to fresh juice samples. The maximum value of total flavonoid (62.29 ± 0.56 mg CE/100 ml) of guava juice was recorded for the thermosonicated sample TS40-2. This increase might be attributed to ultrasonic cavitation rupturing cell walls and releasing these chemicals that are bound to cell walls [48]. Similar result have been observed for the thermosonicated juice of *Meyna spinos*a [19]. Further, at high temperature of 80 °C regardless of processing time, thermosonication reduced the level of flavonoids in comparison to fresh guava juice, but showing higher retention of flavonoid‘s concentration than that of pasteurized guava juice. This might be because high temperature thermosonication reduces the concentration of these chemicals owing to thermal degradation [48]. Moreover, time had a significant effect on the total flavonoid content. It was observed that with an increment in holding time, total flavonoid content was reduced which may be linked to treating variables such power rating, exposure temperature and time [31]. Similar results have been reported for the thermosonicated fruit (*Haematocarpus validus*) juice [35].

### 3.7 Antioxidant activity

Table 2 illustrates the antioxidant activities of the guava juice samples affected by different processing conditions. The antioxidant activity of fresh and pasteurized guava juice samples were 80.9% and 81.45%, respectively and these were non-significantly (p > 0.05) varying with each other. All thermosonicated samples showed an enhancement of 10.45 to 14.55% of DPPH values in comparison to fresh guava juice samples. Similar enhancement of antioxidant activity during thermosonication has been reported for juices of elephant apple (*Dillenia indica*) and blueberry [29], [55]. The increase might be attributed to increased polyphenols caused by cavitation, and this improved the accessibility and extraction of these essential components [26]. Another explanation for the enhanced antioxidant activity might be attributed to the production of hydroxyl radicals by ultrasound part during the hydroxylation of food components, longer sonication exposure may create a significant quantity of hydroxyl radicals in the extract, influencing antioxidant activity negatively [31]. This development may be responsible for slight decrement in the antioxidant values with increasing holding time. Moreover, the various combination of temperature and holding time haven’t significantly (p > 0.05) affected the results of DPPH of guava samples which is similar to findings observed for thermosonicated juice of tangerine juice [5] and sugarcane [2]. The antioxidant capacity of guava juice during thermosonication may have retained due to availability of flavonoids [48] because flavonoid compounds together with procyanidins show high antioxidant capacity due to their structure [3].

### 3.8 Enzymes residual activity

Pasteurized guava juice (PGJ) samples and all thermosonication treatments had substantially (p < 0.05) different impacts on PME, POD, and PPO when compared to untreated guava juice (UGJ) (Table 3). The lowest inactivation of enzyme was observed in juice sample TS40-2 revealing residual activities of PME, POD and PPO as 87.12%, 85.43% and 81.54%, respectively, while the highest inactivation of enzyme was discovered in juice sample TS80-10 indicating residual activities of PME, POD and PPO as 1.92%, 1.45% and 1.03%, respectively. The current study found a smaller decrease in enzyme activity at lower temperatures and larger destruction of enzymes at high exposing temperatures and longer exposing duration. These finding are on par with that observed in thermosonicated juice of pear [40] and blood fruit [35]. The residual activities of PME, POD and PPO in PGJ samples showed non-significant (p < 0.05) difference in comparison to TS60-10 and TS80-2 samples. Thus, indicating that thermosonication has merits over conventional heat treatment by achieving similar enzyme inactivation at lower temperature and avoiding the loss of heat sensitive nutrients. The exertion of mechanical force due to collapse of bubbles during the phenomenon of cavitation has been proposed as one of the main explanation for enzymatic inactivation of foods subjected to ultrasound treatment. Hence, during thermosonication, joint role of temperature and other mechanical forces destroys enzymes by delivering mechanical and thermal shock to these enzymes and consequently altering the proteins’s structure. Ultimately, these crucial environmental factors inactivate enzymes [25]. Thus, juice samples treated with thermosonication produced improved results for enzyme inactivation at various combinations of exposure time and temperature.

**Table 3:** The effect of thermosonication on residual activity percentages of PME, POD and PPO in guava juice.

Treatment	PME	POD	PPO
UGJ	100 ± 1.09^a^	100 ± 0.99^a^	100.00 ± 0.26^a^
PGJ	5.91 ± 1.12^g^	5.02 ± 0.21^g^	4.31 ± 0.01^g^
TS40-2	87.12 ± 0.20^b^	85.43 ± 0.61^b^	81.54 ± 0.89^b^
TS40-6	78.44 ± 0.04^c^	75.21 ± 1.6^c^	68.11 ± 0.91^c^
TS40-10	64.08 ± 0.67^d^	61.32 ± 0.71^d^	54.32 ± 1.18^d^
TS60-2	57.10 ± 0.72^e^	53.25 ± 0.20^e^	49.25 ± 0.91^e^
TS60-6	22.05 ± 0.01^f^	36.10 ± 1.3f	31.10 ± 1.24^f^
TS60-10	6.07 ± 0.20^g^	5.14 ± 0.08^g^	4.87 ± 0.88^g^
TS80-2	5.85 ± 1.15^g^	4.90 ± 1.07^g^	4.09 ± 1.13^g^
TS80-6	2.01 ± 1.08^h^	1.72 ± 0.06^h^	1.51 ± 0.34^h^
TS80-10	1.92 ± 0.03^h^	1.45 ± 0.04^h^	1.03 ± 0.08^h^

*Note*: Means ± SD, Values with same superscript letters in the same column are non-significant at p < 0.05.

### 3.9 Microbial analysis

**Fig. 2:**
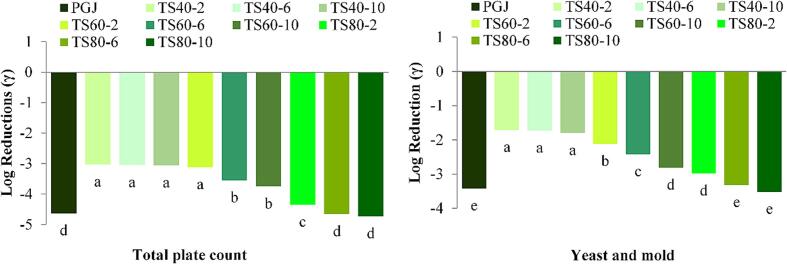
Log reductions (γ) of total plate count and yeast and mold of guava juice treated by different conditions of thermosonication. Bars with same superscript letters are non-significant at p < 0.05.

Fruit juices, due to their high nutrient content, provide an ideal environment for microorganisms to thrive, raising the risk of foodborne infections and their associated dangers [52]. Fig. 2 shows the log reduction of pasteurization and thermosonication. The PGJ sample showed near to 5 log reductions of total plate count and near to 3.5 log reduction of yeast and mold count. This inactivation might be associated with pasteurization causing the cell wall/membrane and nuclear components to rupture and leading to cell death [31]. Likewise, the microbial load in chokananan mangao juice was reduced to levels below the detection limit during thermal pasteurization as reported by Santhirasegaram et al. [42]. Similarly, thermosonication significantly (p < 0.05) achieved microbial decontamination of guava juice as shown in Fig. 2. At processing temperature of 40 °C, a 3.03, 3.05 and 3.03 log reductions in the total plate count was observed after thermosonication of 2, 6 and 10 min, respectively, while 1.70–1.80 log reductions were reported for yeast and mold as the treatment time was increased from 2 to 10 min. This slight increase observed in microbial inactivation with enhancement of holding time at 40 °C might be linked to spore development, which could withstand such treatment conditions [25]. An enhanced microabial inactivation was observed at 60 °C where the 2–10 min of treatment reduced the total plate count by 3.12–3.75 log cycles and yeast and mold count by 2.12–2.81. Further, a 4.35 to 4.73 log reduction of total plate count and 2.98 to 3.52 log reduction of yeast and mold at temperature of 80 concluded that inactivation of microbial population was more pronounced with longer treatment times and higher temperature. This is mainly attributable to the cumulative impact caused by cavitation combined with elevated temperature [2]. Similar microbial inactivation have been reported in tomato juice and orange juice whey drink [20], [32]. It can be observed that the highest log reduction of yeast and mold count (3.52) was lesser than higest log reduction (4.73) of total plate count inactivation. Yeast and mold displayed greater resistance to ultrasound compared to bacteria, possibly because of differences in cell wall thickness [16]. The inactivation mechanism of microorganisms through thermosonication is the result of numerous complex physical processes [26]. The mechanical breakdown of microbial cells by ultrasound is the main factor behind its inhibitory impact on these cells. The breakdown of microbial cell walls is the end outcome of cavitation and mechanical impact caused by large pressure differences [14]. Additionally, the sonolysis of water generate free radicals (H^+^, O^.^_2_, OH^.^, HOO^.^) which causes oxidative damage in microbial cells [8].

### 3.10 Sensory analysis

The sensory score, which indicates acceptance of end user to untreated and processed juice, revealed that untreated juice scored marginally superior than pasteurized juice as shown in Table 4. Color, flavor, tongue feel, and overall acceptability scores in fresh guava juice were 8.12, 8.10, 7.98, and 8.05, respectively and that for pasteurized juice were 7.98, 7.94, 7.86 and 7.91, respectively. It was observed that the sensory score of pasteurized juice was not only lower than fresh juice but also lower than all the thermosonicated samples. The reduced overall sensory acceptance could be attributed to pasteurization, which might have caused alteration in taste due to production of undesirable flavors and degradation of flavor compounds, change in color due to non-enzymatic browning, and pigment loss [23] and variation in mouthfeel due to deterioration of certain compounds which gives unique mouthfeel to guava juice [27], [28]. On the other hand, higher sensory scores of color (8.15–8.55), taste (8.15–8.51), mouthfeel (8.07–8.51) and overall acceptability (8.17–8.50) were reported for the samples thermosonicated at 40 and 60 °C when compared with fresh juice samples. Cavitation, induced by thermosonication treatments, could be associated with the reduction of oxygen content in fruit juice, potentially resulting in improved sensory properties [57]. Additionally, the sensory improvements observed with thermosonication may be attributed to enhanced filterability, a lighter juice color, reduced enzyme activity (specifically pectin methyl esterase (PME)), and higher retention of ascorbic acid [54]. Similar increase in the sensory score have been observed in hog plum juice treated with thermosonication [31]. On contrary, prolonged treatment of 10 min at an elevated temperature of 80 °C decreased scores of color (8.00), taste (7.99), mouthfeel (7.89) and overall acceptability (7.96) which in accordance with results observed in thermosonicated fruit (*Haematocarpus validus*) juice [46]. The extended exposure to high temperatures during thermosonication treatment might have adversely impacted the juice's sensory characteristics by potentially deteriorating certain fresh flavor compounds and introducing cooked notes [49].

**Table 4:** The effect of different conditions of thermosonication on sensory attributes of guava juice.

Treatment	Color	Taste	Mouth feel	Overall acceptability
UGJ	8.12 ± 0.07^a^	8.10 ± 0.06^a^	7.98 ± 0.09^a^	8.06 ± 0.33^a^
PGJ	7.98 ± 0.20^a^	7.94 ± 0.34^a^	7.86 ± 0.33^a^	7.92 ± 0.35^a^
TS40-2	8.30 ± 0.26^a^	8.15 ± 0.10^a^	8.07 ± 0.30^a^	8.17 ± 0.13^a^
TS40-6	8.40 ± 0.07^a^	8.28 ± 0.02^a^	8.19 ± 0.01^a^	8.29 ± 0.19^a^
TS40-10	8.50 ± 0.26^a^	8.42 ± 0.04^a^	8.34 ± 0.30^a^	8.42 ± 0.19^a^
TS60-2	8.55 ± 0.21^a^	8.50 ± 0.09^a^	8.42 ± 0.36^a^	8.50 ± 0.35^a^
TS60-6	8.37 ± 0.37^a^	8.51 ± 0.13^a^	8.51 ± 0.27^a^	8.46 ± 0.03^a^
TS60-10	8.25 ± 0.16^a^	8.48 ± 0.29^a^	8.41 ± 0.28^a^	8.38 ± 0.07^a^
TS80-2	8.19 ± 0.26^a^	8.29 ± 0.31^a^	8.24 ± 0.31^a^	8.24 ± 0.21^a^
TS80-6	8.15 ± 0.34^a^	8.15 ± 0.34^a^	8.12 ± 0.32^a^	8.14 ± 0.05^a^
TS80-10	8.00 ± 0.29^a^	7.99 ± 0.01^a^	7.89 ± 0.34^a^	7.96 ± 0.34^a^

*Note*: Means ± SD, Values with same superscript letters in the same column are non-significant at p < 0.05. GAE = Gallic acid equivalent.

## 4 Conclusion

In this study, thermosonication (TS) of fresh was done as a strategy to improve overall quality and thus improving its economic worth over conventional processing method. The TS preserved the guava juice's quality attributes, primarily by improving the value of cloud and color attributes. TS not only retained high content of ascorbic acid but also enhanced the levels of total phenols, flavonoids and antioxidant activity. TS significantly inactivated the loads of enzymes (PME, POD, PPO) and microbes (total plate count and yeast and mold) with slight enhancement of sensorial properties. The quality enhancement of guava juice can easily be attained with the application of TS. In comparison to thermal processing, the present investigation reveals that TS is a suitable technology for processing of guava juice. Furthermore, the shelf-life of the thermosonicated guava juice during storage is subject to future examination in order to determine its economic benefits employing this technology.

## CRediT authorship contribution statement

**Baldev Singh Kalsi:** Investigation, Conceptualization, Methodology, Formal analysis, Software, Writing – original draft, Data curation, Writing – review & editing. **Sandhya Singh:** Conceptualization, Methodology, Supervision, Project administration, Validation, Writing – review & editing. **Mohammed Shafiq Alam:** Software, Data curation, Formal analysis, Writing – review & editing. **Surekha Bhatia:** Formal analysis, Writing – review & editing.

## Declaration of Competing Interest

The authors declare that they have no known competing financial interests or personal relationships that could have appeared to influence the work reported in this paper.
